# A fast robust optimizer for intensity modulated proton therapy using GPU

**DOI:** 10.1002/acm2.12835

**Published:** 2020-03-06

**Authors:** Yao Xu, Jinhu Chen, Ruifen Cao, Hongdong Liu, Xie George Xu, Xi Pei

**Affiliations:** ^1^ School of Physical Sciences University of Science and Technology of China Hefei Anhui China; ^2^ Department of Radiation Oncology Shandong Tumor Hospital and Institute Jinan Shandong China; ^3^ School of Computer Science and Technology Anhui University Hefei Anhui China; ^4^ Nuclear Engineering Program Rensselaer Polytechnic Institute Troy NY USA

**Keywords:** GPU, IMPT, proton pencil beams, robust optimization, the conjugate gradient method

## Abstract

Robust optimization has been shown to be effective for stabilizing treatment planning in intensity modulated proton therapy (IMPT), but existing algorithms for the optimization process is time‐consuming. This paper describes a fast robust optimization tool that takes advantage of the GPU parallel computing technologies. The new robust optimization model is based on nine boundary dose distributions — two for ±range uncertainties, six for ±set‐up uncertainties along anteroposterior (A‐P), lateral (R‐L) and superior‐inferior (S‐I) directions, and one for nominal situation. The nine boundary influence matrices were calculated using an in‐house finite size pencil beam dose engine, while the conjugate gradient method was applied to minimize the objective function. The proton dose calculation algorithm and the conjugate gradient method were tuned for heterogeneous platforms involving the CPU host and GPU device. Three clinical cases — one head and neck cancer case, one lung cancer case, and one prostate cancer case — were investigated to demonstrate the clinical feasibility of the proposed robust optimizer. Compared with results from Varian Eclipse (version 13.3), the proposed method is found to be conducive to robust treatment planning that is less sensitive to range and setup uncertainties. The three tested cases show that targets can achieve high dose uniformity while organs at risks (OARs) are in better protection against setup and range errors. Based on the CPU + GPU heterogeneous platform, the execution times of the head and neck cancer case and the prostate cancer case are much less than half of Eclipse, while the run time of the lung cancer case is similar to that of Eclipse. The fast robust optimizer developed in this study can improve the reliability of traditional proton treatment planning in a much faster speed, thus making it possible for clinical utility.

## 1 Introduction

Owing to the steep distal dose gradient of proton beams, intensity modulated proton therapy (IMPT) is known to deliver more conformal dose distributions compared with those of photon therapy.^1^ For IMPT treatment planning, the target volumes are divided into separate energy slices according to the Bragg peak positions corresponding to different incident proton beam energies.^2^ There are many procedures that could cause uncertainties in IMPT, such as conversions from computed tomography (CT) numbers to stopping power values, inhomogeneity, setup errors, anatomical changes, and so on.^3^, ^4^, ^5^, ^6^, ^7^, ^8^ As a result, the actual delivered dose distribution of IMPT plan will be affected by these uncertainties. Scanning spots or scanning lines are preset and optimized to meet the dose objectives and constraints in the inverse treatment plan. However, due to the steep falloff of Bragg peaks, IMPT is extremely sensitive to proton range and patient setup uncertainties. For photon therapy, clinical target volume (CTV) is expanded to the planning target volume (PTV) by a given margin to compensate for plan uncertainties, but the PTV expanding technique is not as effective in IMPT.^9^

The solution of the above problems is to consider these uncertainties during the inverse treatment plan optimization — a process known as the robust optimization.^10^ Several related reports for robust optimization of IMPT have shown better results than common PTV‐based IMPT optimization techniques. Unkelbach et al.^6^ proposed a robust linear programing method that was found to yield treatment plans less sensitive to range variations. A worst case optimization method was developed by Pflugfelder et al.^10^ to account for uncertainties based on several possible realizations of the uncertainties A minimax robust optimization method was utilized by Fredriksson et al.^11^ to minimize the penalty of the worst scenario which yielded better results for the tested clinical cases. Generally, in worst‐case‐based robust algorithms, the nominal dose distribution and perturbed dose distributions are calculated. When computing the target function, upper bound constraints are applied to the maximum dose while lower bound constraints are applied to the minimum dose.^12^, ^13^, ^14^, ^15^, ^16^, ^17^ A selective robust optimization method was derived by Li et al.^18^ from worst case optimization methods and its objective function was selectively computed from either the worst‐case dose or the nominal dose. Li et al.^19^ reported that robust optimization in IMPT of lung cancer can reduce the dose variation caused by setup uncertainty and anatomical changes during treatment compared with PTV‐based planning. Although three worst‐case‐based robust methods (composite worst case, voxelwise worst case and objectivewise worst case) have different behaviors, and no particular method was superior to the others under all circumstances.^20^ Compared with minmax robust optimization approaches, worst‐case dose approaches were less sensitive to uncertainties for the prostate and skull base cancer patients, whereas the minmax approach was superior for the head and neck cancer patients.^21^ A 4D robust optimization was developed by Liu et al.^22^ and it produced more robust plans for targets and normal tissues, compared to 3D robust optimization. In order to accelerate the robust optimization, a constraint generation solution method was developed by Mahmoudzadeh et al,^23^ which reduced the optimization time to about 12 min. A chance‐constrained optimization method was proposed in IMPT planning to hedge against the influence of uncertainties,^24^ but several hours were needed to finish the optimization. The chance‐constrained optimization method explicitly controlled the tradeoff between plan quality and plan robustness, and it used linear programing without parallelization, making the method very slow. Jiasen Ma et al developed an all scenario and MC‐based IMPT optimizer and employed GPU to reduce the computational time.^25^

In this research, we propose the uncertainty model which contains nine boundary dose distributions corresponding to different setups and range perturbations, two for ±range uncertainty, six for ±set‐up uncertainties along anteroposterior (A‐P), lateral (R‐L) and superior‐inferior (S‐I) directions, and one for the nominal situation. All the nine dose distributions consider the target function and optimized using the same optimization objective in each optimization iteration until all the nine dose distributions approach the dose constraints as much as possible. In order to reduce the optimization time, the proposed method is implemented in a CPU‐GPU parallel platform. Three clinical cases are used to test the robust optimization effectiveness.

## 2 Materials and Methods

The proposed uncertainty model contains mathematical descriptions of range uncertainties and patient setup errors based on the standard optimization function [Eq. (1)]. The pencil beam algorithm is used to calculate the proton dose contribution matrices, and the conjugate gradient (CG) method is used to optimize the uncertainty model. This paper evaluates the effectiveness of the proposed robust optimizer for IMPT by comparing against the robust optimization results of Varian Eclipse (version 13.3).

### 2.1 The uncertainty optimization model

For the traditional PTV‐based IMPT optimization model, the standard quadratic objective function can be used to represent optimization objectives and constraints similar to IMRT.^26^ Mathematically speaking, treatment plan optimization is the minimization of the objective function and is given below by:

(1) $$\begin{array}{c} \begin{array}{c} \min F\left(\omega \right)=\sum_{i\;\in \;\text{PTV}}\rho_{\text{PTV}}H\left(D_{i}-D_{\text{PTV}}\right){\left(D_{i}-D_{\text{PTV}}\right)}^{2}\\ \;+\sum_{i\;\in \;\text{OARs}}\rho_{\text{OARs}}H\left(D_{i}-D_{\text{OARs}}\right){\left(D_{i}-D_{\text{OARs}}\right)}^{2} \end{array} \end{array}$$

(2) $$D_{i}=\sum_{j=1}^{m}\varphi_{i,j}\cdot \omega_{j}$$

(3) $$\omega_{j}\geq 0,\forall j\in m,$$

where *i* is the index of voxel *i*, *j* is the proton beamlet *j* and *m* is the total number of the proton beams. The weights of PTV and OARs were represented by $\rho_{\text{PTV}}$, and $\rho_{\text{OARs}}$, respectively. $D_{i}$ is the dose of the *i*th voxel, and $\varphi_{i,j}$ is the dose contribution of the *j*th beam to the *i*th voxel computed by the pencil beam dose calculation algorithm. $\omega_{j}$ is MU (Monitor Unit) of the *j*th proton beam. $D_{\text{PTV}}$ and $D_{\text{OARs}}$ represent the dose objectives or constraints of the PTV and OARs, and $H\left(D_{i}-D_{\text{PTV}}\right)$ is a selection function. When $D_{i}$ satisfies the dose objective or constrain of the PTV, the $H\left(D_{i}-D_{\text{PTV}}\right)$ value is equal to 0; otherwise it equals 1. $H\left(D_{i}-D_{\text{OARs}}\right)$ is the same as $H\left(D_{i}-D_{\text{PTV}}\right)$. For dose volume constraints, *H* is determined by sorting the doses, finding violating voxels. For example, $D_{q}<p\;\text{cGy}$ means no more than q% of the ROI may receive a dose greater than p. The voxels of the ROI are sorted in ascending order of dose received and only those voxels which cause q to be exceeded are included in the objective function (H = 1).^27^ As mentioned earlier, $\varphi_{i,j}$ is perturbed by range and setup uncertainties impacting on the finally dose contributions of the PTV and OARs. For the IMPT plan delivery, the final dose distribution is affected by many factors including complex inhomogeneity, setup errors, anatomical changes, and so on.^3^, ^4^, ^5^, ^6^, ^7^, ^8^ All these interference factors will result finally in two kinds of deviations relative to the primary plan, proton range uncertainties, and patient set‐up uncertainties. Compared to the traditional plan optimization, robust plan optimization takes the two kinds of deviations into consideration beforehand. The challenge is that, for IMPT robust optimization, the computational complexity increases proportionally to the number of potential of considered uncertainties. In theory, more uncertainties can lead to better robust plan during robust optimization. Considering the large computation cost even for one possible situation, however, we have to balance between the uncertainties taken into consideration and the actual performance of robust optimization. As reported previously^12^, ^13^, the simplification for IMPT robust optimization is usually handled in two processes. The first simplification process is discretizing the proton range uncertainties and patient set‐up uncertainties before selecting the extreme uncertainties as a boundary. In this study, we use the same discretizing method to simplify the robust optimization task, as to be shown next. The second simplification is that only the worst dose distributions are included in the target function. Thus, we can only get a relatively conservative result due to the robust optimization simplification. In this research, we improved the target function by considering all distributions in the total target function so that not merely the worst distribution but all the dose distributions approached the dose constrains. The CPU‐GPU parallel platform was further adopted to increase computational efficiency, as to be illustrated next.

The proposed robust optimization model adopted in this research is shown in Eq. (4) in which nine boundary dose distributions are considered, two for ±range uncertainties, six for ±set‐up uncertainties along anteroposterior (A‐P), lateral (R‐L) and superior‐inferior (S‐I) directions, and one for nominal situation.

(4) $$\begin{array}{cc} \min\;F_{\text{robust}}\left(\omega_{j}\right) & =\sum_{k\;\in \;R}\sum_{i\;\in \;\text{CTV}}\rho_{\text{CTV}}^{k}H\left(D_{i}^{k}-D_{\text{CTV}}\right){\left(D_{i}^{k}-D_{\text{CTV}}\right)}^{2}\\ & \;+\sum_{k\;\in \;R}\sum_{i\;\in \;\text{OARs}}\rho_{\text{OARs}}^{k}H\left(D_{i}^{k}-D_{\text{OARs}}\right){\left(D_{i}^{k}-D_{\text{OARs}}\right)}^{2} \end{array}$$

(5) $$D_{i}^{k}=\sum_{j=1}^{m}\varphi_{i,j}^{k}\cdot \omega_{j}$$

(6) $$\omega_{j}\geq 0,\forall j\in m,$$

where *R* is the total nine boundary dose distributions considering the range and setup uncertainties, and *k* represents one possible boundary dose distributions of *R*. $\rho_{\text{CTV}}^{k}$ and $\rho_{\text{OARs}}^{k}$ are the weights of CTV and OARs in the *kth* uncertainty situation, usually equal to 1. $D_{i}^{k}$ is the dose of voxel *i* in the *kth* uncertainty, and $\varphi_{i,j}^{k}$ is the dose contribution of beamlet *j* to voxel *i* in the *kth* uncertainty situation. The objective function is minimized by optimizing $\omega_{j}$. It is impossible to forecast which uncertainty will occur in the treatment, so it is necessary to ensure that the treatment planning is optimal on the premise of the existence of the nine boundary conditions.

As the same with the reported researches,^12^ we made the assumptions: (1) all the beamlets were affected by the range uncertainties and changed synchronously; (2) the max range uncertainties and setup uncertainties value kept the same during the treatment course.

### 2.2 The robust optimization method

**Figure 1:**
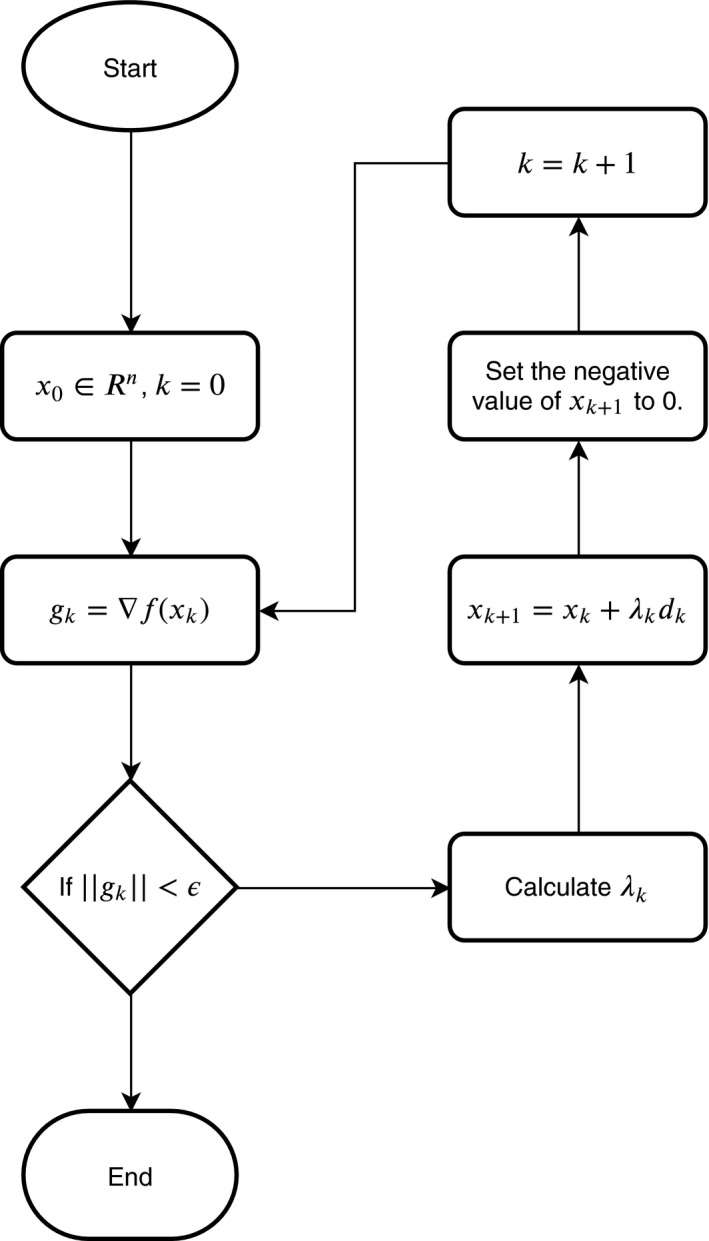
Flow chart of the conjugate gradient method.

The CG method is one of the most popular techniques for solving large scale unconstrained optimization problems owing to small memory footprint and simplicity. Figure 1 demonstrates the flow chart of the CG method. $x_{0}$ is the initial solution, and *k* is the current iteration number. *g_k_* is the gradient of *x_k_*, ε is the tolerance to stop the iterative process and *N* is the maximum number of iterations. *d* is the searching direction, and λ is the step length.

In the first iteration, the searching direction *d*_0_ is equal to the negative gradient, that is $\nabla f\left(x_{0}\right)$. For other iterations, $d_{k}$ is defined by.

(7) $$d_{k}^{i}=\left\{\begin{array}{cc} -\frac{x_{k}^{i}}{\lambda_{k}}, & if\;x_{k}^{i}+\lambda_{k}\cdot d_{k}^{i}<0\\ -g_{k}^{i}+\beta_{k-1}^{i}d_{k-1}^{i}, & \text{otherwise;} \end{array}\right.,$$

where $\beta_{k}$ is a scalar. It guarantees that all elements of $x_{k+1}$ are greater than 0.

In this paper, the DYHS method [Eq. (8)] is adopted to calculate the CG parameter β.

(8) $$\beta_{k}^{\text{DYHS}}=\max\left\{0,\min\left(\beta_{k}^{\text{DY}},\beta_{k}^{\text{HS}}\right)\right\}$$

(9) $$\beta_{k}^{\text{DY}}=\frac{\left|\right|g_{{}_{k-1}}{\left|\right|}^{2}}{d_{k-1}^{T}\left(g_{k}-g_{k-1}\right)}$$

(10) $$\beta_{k}^{\text{HS}}=\frac{g_{k}^{T}\left(g_{k}-g_{k-1}\right)}{d_{k-1}^{T}\left(g_{k}-g_{k-1}\right)}$$

The DYHS method is a combination of the DY method and the HS method.^28^, ^29^, ^30^

The line search subroutine yields a step length $\lambda_{k}$ that satisfies the standard Wolfe conditions [Eqs. (11) and (12)],

(11) $$f\left(x_{k}+\lambda_{k}d_{k}\right)\leq f\left(x_{k}\right)+\lambda_{k}\delta g_{k}^{T}d_{k},$$

(12) $$g{\left(x_{k}+\lambda_{k}d_{k}\right)}^{T}d_{k}\geq \theta g_{k}^{T}d_{k},$$

where $g_{k}=\nabla f\left(x_{k}\right)$, $\delta =10^{-4}$, and θ=0.9. The first inequation is to guarantee that the decrease of the target function is proportional to the tangential decline at least. The second inequation ensures the slope at $\lambda_{k}$ is not strongly negative.

### 2.3 Heterogeneous platform with CPU and GPU

**Figure 2:**
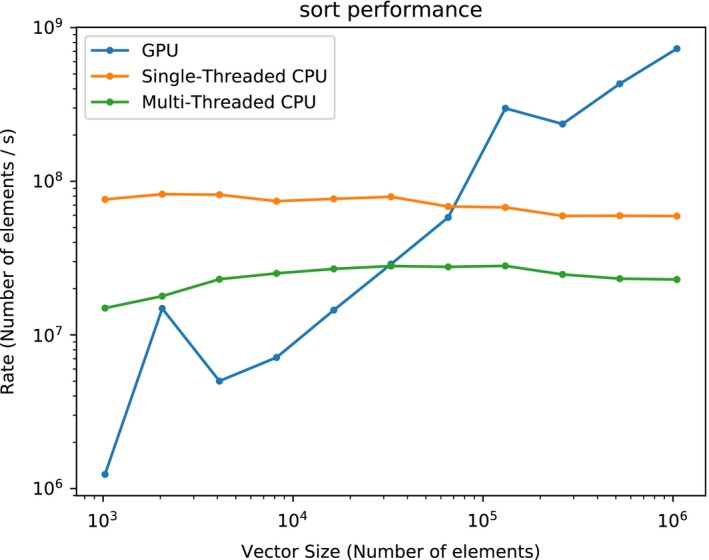
Comparison between CPU and GPU sort performance.

A GPU dose engine is adopted to calculate the dose contribution matrix $\varphi_{i,j}$ and then the matrix is converted to the most memory efficient sparse matrix format, that is compressed sparse row (CSR) format.^31^ The CSR format uses three arrays to store the nonzero elements, corresponding column indices and compressed row offsets which indicate the boundary of each row. The most important computing kernels of the proposed optimizer are sparse matrix dense vector product (SpMV), gradient calculation, and vector operations. The SpMV algorithm proposed by bell and Garland is applied to update dose vector $D^{k}$ and modified to support gradient calculation.^32^ Offloading SpMV and gradient calculation to GPU can always improve the performance. However, the CPU version of target value calculation which involves vector operations outperforms the GPU version when the number of voxels is relatively small. As mentioned earlier, the doses of corresponding ROIs need to be sorted before computing the objective function of dose‐volume constraints. Figure 2 demonstrates the sort performance of CPU (E5 2686V3) and GPU (NVIDIA Titan V). The single‐threaded version is fastest when the vector size is smaller than about 6 × 10^4^. Other vector operations follow the similar performance patterns.

### 2.4 Patient data

Three cases, one head and neck (H&N) case, one lung case and one prostate case, are used to test the effectiveness of the proposed optimizer. For the H&N case treatment plan, the three beams angels were set to 0°, 90°, and 270°. Setup uncertainties of ±3 mm and range uncertainties of ±3% relative to the beams' nominal ranges were assumed. For the lung case treatment plan, the two beams angels were set to 60° and 320°. Setup uncertainties of ±5 mm and range uncertainties of ±3% relative to the beams' nominal ranges were assumed. For the prostate case treatment plan, the two beams angels were set to 90° and 270°. The range and setup uncertainties were assumed the same as the lung case. The spot space in each energy slice was 5 mm and was distributed in BEV. The spacing between energy layers was set to the longitudinal width of the Bragg peak (at 80% of the peak height). Dose grid was set to 2 mm × 2 mm in the transverse plane. Dose objectives and constrains are listed in Table 1. Since the uncertainties had been taken into consideration during the robust optimization, we prescribed the dose objectives based on the CTV.^12^ The volume of the target of the H&N case, the lung case and the prostate case are 51.6, 97.7, and 854.4 cm^3^, and the voxel number of target and OARs in the H&N case, the lung case, and the prostate case are 159882, 4600764, and 389493 (all nine conditions are included). The spots number of the H&N case, the lung case, and the prostate case are 19978, 11844, and 99408.

**Table 1:** Dose objectives and constraints of the three cases.

Object	Constraint
*Head & neck case*	
Target	$D_{\min}>7000\;\text{cGy}$, $D_{\max}<7800\;\text{cGy}$
Brainstem	$D_{\max}<5000\;\text{cGy}$
Parotid‐l	$D_{\max}<3000\;\text{cGy}$
Parotid‐r	$D_{\max}<3000\;\text{cGy}$
Spinal Cord	$D_{\max}<4000\;\text{cGy}$
*Lung case*	
Target	$D_{\max}<6400\;\text{cGy}$, $D_{\min}>6000\;\text{cGy}$
Dose 64	$D_{\max}<6200\;\text{cGy}$
Esophagus	$D_{54}<3300\;\text{cGy}$
Spinal Cord	$D_{\max}<3500\;\text{cGy}$
Fan	$D_{\max}<3500\;\text{cGy}$
Lung‐l	$D_{2.8}<138\;\text{cGy}$, $D_{1.2}<721\;\text{cGy}$
Lung‐r	$D_{8.5}<249\;\text{cGy}$, $D_{4.6}<1343\;\text{cGy}$
*Prostate case*	
Target	$D_{\max}<5300\;\text{cGy}$, $D_{1}<5280\;\text{cGy}$
	$D_{98.5}>5050\;\text{cGy}$, $D_{99.5}>5000\;\text{cGy}$
Rectum	$D_{54.4}<2664\;\text{cGy}$, $D_{26.5}<3692\;\text{cGy}$
Bladder	$D_{42.3}<2049\;\text{cGy}$, $D_{20.9}<3097\;\text{cGy}$
Head of femur‐l	$D_{43.9}<1219\;\text{cGy}$, $D_{15.2}<1754\;\text{cGy}$
Head of femur‐r	$D_{41.7}<1302\;\text{cGy}$, $D_{16.9}<1702\;\text{cGy}$

## 3 Results

To test the effectiveness of the robust optimizer, the results were compared with Varian Eclipse (version 13.3). The robustness of the treatment plan was quantified by computing the worst‐case dose distribution for each approach. Dose Volume Histograms (DVHs) for the reference structures of interest corresponding to different uncertainty situations (e.g., setup, range) were demonstrated along with the DVH in the nominal situation. The “band” of the DVHs showed the robustness of the treatment plan when considering uncertainties, and the wider DVH band meant the greater plan sensitivity.

The proposed optimizer was deployed to a workstation consisting of Intel Xeon 2686V3 (18 cores, 36 threads), 64.0GB DDR4 2133 MHz (quad‐channel), and NVIDIA Titan RTX while the Varian workstation had 2 Intel Xeon E5 2620V3 (12 cores, 24 threads), 32.0GB DDR4 2133 MHz, and NVIDIA M4000. Varian Eclipse (version 13.3) did not support GPU acceleration.

**Figure 3:**
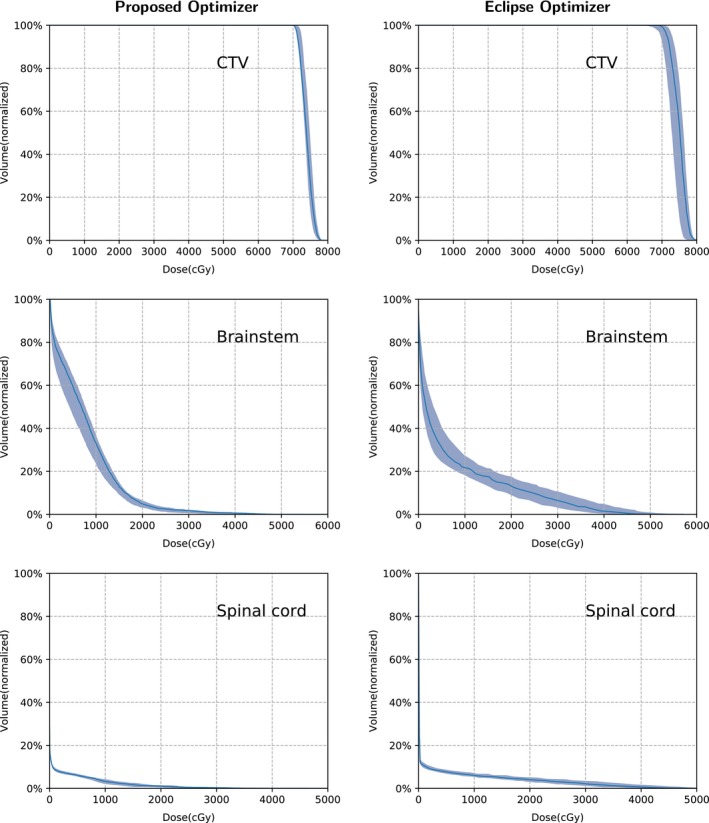
The Dose Volume Histogram (DVH) bands of the dose distributions considering uncertainties for the H&N case with the solid lines indicating the nominal dose distribution.

**Figure 4:**
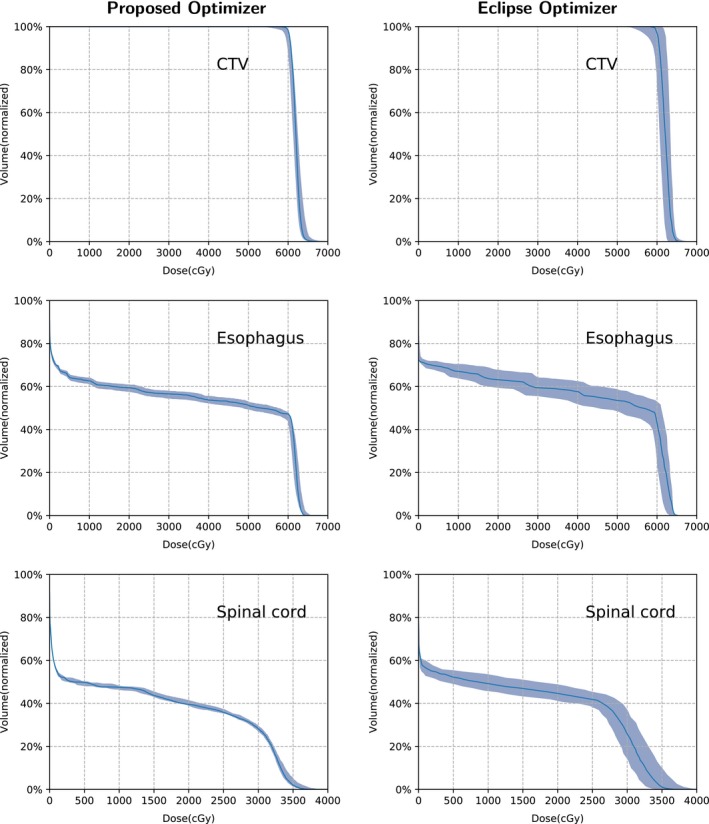
The Dose Volume Histogram (DVH) bands of the dose distributions considering uncertainties for the lung case with the solid lines indicating the nominal dose distribution.

**Figure 5:**
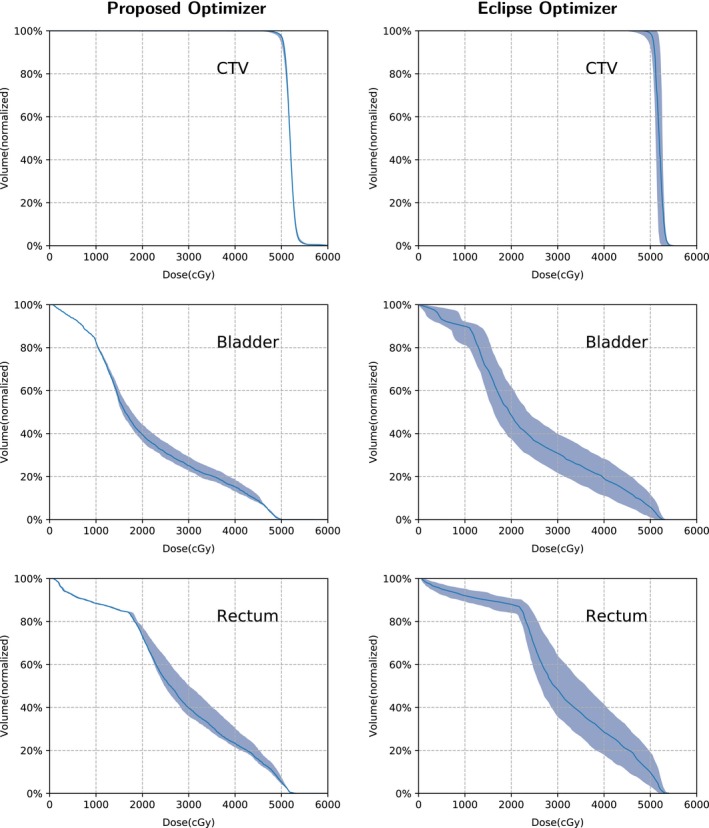
The Dose Volume Histogram (DVH) bands of the dose distributions considering uncertainties for the prostate case with the solid lines indicating the nominal dose distribution.

**Figure 6:**
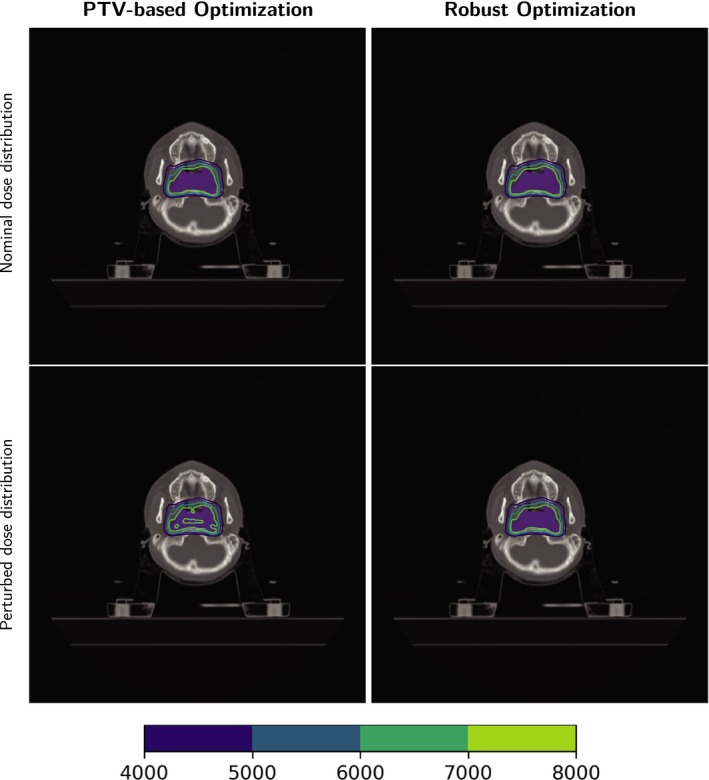
Dose distributions in the transverse plane for the H&N case. Left panels: PTV‐based optimization. Right panels: robust optimization. Top row: with nominal range and nominal position. Bottom row: with 3% range overshoot and patient shifted inferiorly by 3 mm. CTV: purple color wash.

**Figure 7:**
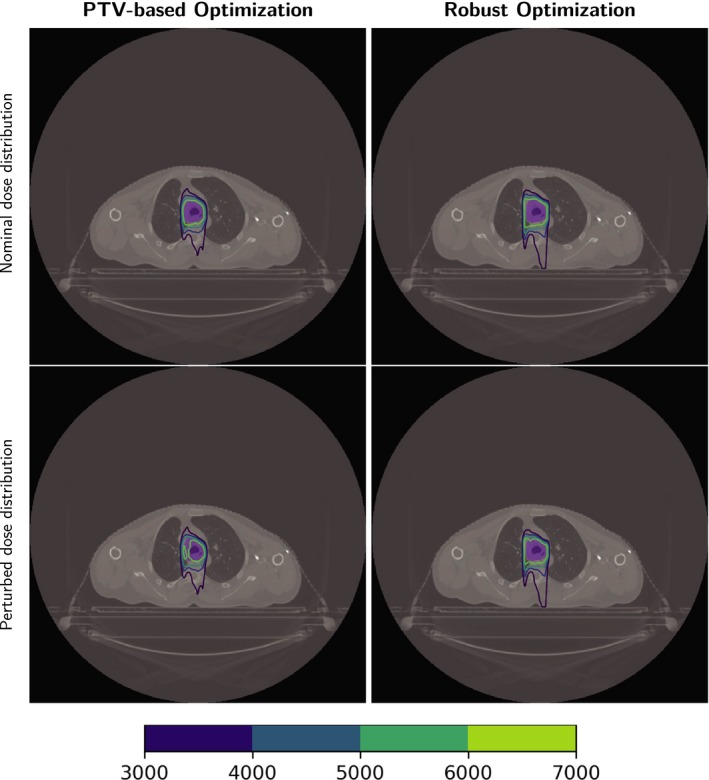
Dose distributions in the transverse plane for the lung case. Left panels: PTV‐based optimization. Right panels: robust optimization. Top row: with nominal range and nominal position. Bottom row: with 3% range overshoot and patient shifted inferiorly by 3 mm. CTV: purple color wash.

**Figure 8:**
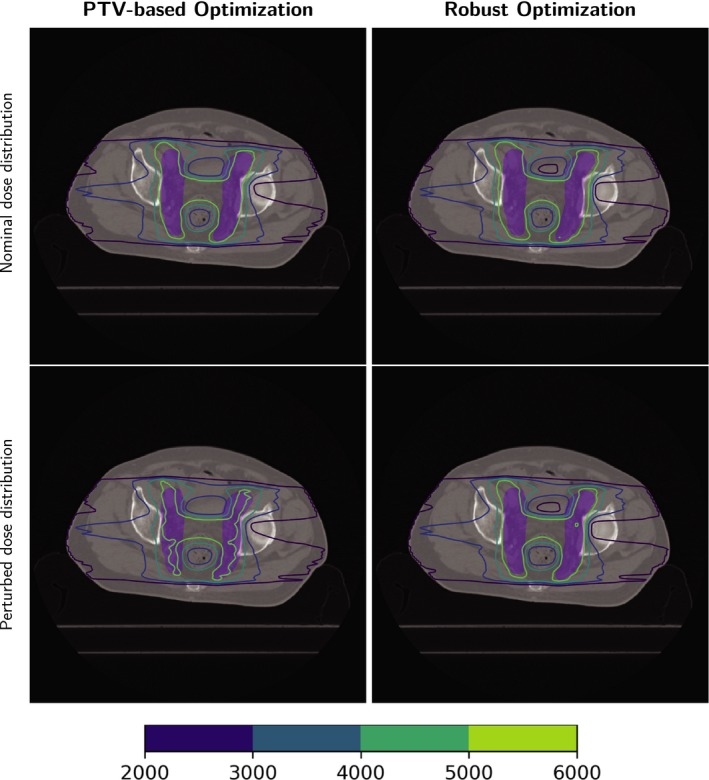
Dose distributions in the transverse plane for the prostate case. Left panels: PTV‐based optimization. Right panels: robust optimization. Top row: with nominal range and nominal position. Bottom row: with 3% range overshoot and patient shifted inferiorly by 3 mm. CTV: purple color wash.

Table 2 lists the run times of the three cases (the times of dose calculation are not included). Figure 3 shows the DVH bands of the CTV, brain stem, and spinal cord for the head and neck cancer case; Fig. 4 shows the DVH bands of the CTV, esophagus, and spinal cord for the lung case and Fig. 5 shows the DVH bands of the CTV, bladder, and rectum for the prostate case. Obviously, the DVH bands of the proposed optimizer are narrower than Varian Eclipse's, indicating that the proposed optimizer is less sensitive to setup and range uncertainties. In addition, the proposed optimizer achieves better OAR protection. For example, the maximum dose of the brainstem of the proposed optimizer is much lower than the maximum dose of Varian Eclipse. Figures 6, 7, 8 demonstrate the transverse dose distributions of the H&N case, lung case, and prostate cased. It is clear that dose distributions of robustly optimized plans are less sensitive to range and setup uncertainties compared with PTV‐based plans.

**Table 2:** The run times of the three cases (seconds).

	Proposed optimizer (CPU)	Proposed optimizer (GPU)	Eclipse optimizer (CPU)
Head & neck	16	3	75
Lung	105	38	48
Prostate	218	26	474

## 4 Discussion

Compared with photon radiotherapy, the physical properties of proton beam (steep Bragg peak) determine that the proton dose distributions are more sensitive to the uncertainties. For IMPT, the treatment plan obtains the desired dose distribution for the target and the organs at risk by optimizing weight of the proton spots on the multiple energy layers. It is necessary to consider the impact of uncertainties when evaluating the proton radiotherapy plans. In this paper, the worst dose distribution considering the range and setup uncertainties was calculated as a criterion to evaluate the robustness of proton radiotherapy plans. This paper proposed a fast robust optimizer, which considering the nominal range, increased and shortened range and setup uncertainties.

For the H&N case, the desired dose of CTV is less than 7800 cGy. The worst plan generated by the proposed optimizer nearly meets the constraint with the maximum dose 7827 cGy while the worst plan of Varian Eclipse exceeds the constraint with the maximum dose 8195 cGy. The maximum dose of the spinal cord in worst plan of Varian Eclipse is 4850 cGy while the maximum dose generated by the proposed optimizer is 3992 cGy. For the lung case, the worst plan of Varian Eclipse does not satisfy the constraint of the spinal cord $\left(D_{\max}<3500\;\text{cGy}\right)$ with the maximum dose 3691 cGy. Besides, for the prostate case, the DVH bands of the proposed optimizer are much narrower than Varian Eclipse's.

The objective function of the proposed optimizer is a function of all nine dose distributions. At each iteration, the proposed optimizer involves nine uncertainty conditions rather than only the worst condition. Consequently, the memory footprint is much larger than commercial treatment planning systems. In contrast, the objective function of Eclipse is a function of the nominal, the minimum, the maximum dose distributions. In fact, Eclipse consumed less than 3GB memory for all cases while the proposed optimizer used more than 10GB. Although the proposed optimizer consumed huge memory, Figs. 3, 4, 5 indicates it can generate plans that are less sensitive to uncertainties and have better OAR protection. From Table 2, it means that the performance of the proposed optimizer is highly competitive against Varian Eclipse. For the H&N case, the GPU version of the proposed optimizer is 25 times faster than Varian Eclipse. For the lung case, the proposed optimizer is slightly faster than Varian Eclipse. For the prostate case, the GPU version of the proposed optimizer is 18 times faster than Varian Eclipse. For the H&N case and the lung case, the GPU version achieves approximately 3× speedup than the CPU version.

The performance bottleneck of the proposed optimizer is SpMV which is used to update the dose and gradient vectors. Given the memory bound property of SpMV, system bandwidth is the most important hardware specification. The bandwidth of quad‐channel DDR4 2133 MHz is around 60GB/s while Titan V peaks around 500 GB/s. However, PCIe 3.0 × 16 has about 16 GB/s which limits data transfer between CPU and GPU. Accordingly, dose contribution matrix should not be accessed across the PCIe slot in the iterative process. Additionally, technologies such as CUDA unified memory may cause performance downgrade. In general, the robust proposed optimizer can exploit high memory bandwidth of GPU to accelerate SpMV and fall back to CPU with high memory capacity for extreme cases.

## 5 Conclusions

The proposed robust optimizer can greatly improve the robustness of the proton plan to compensate for the range and setup uncertainties. Compared with the Varian Eclipse (version 13.3), the proposed fast robust optimizer can improve the high dose uniformity of the target, meanwhile, protect the OARs. Based on a CPU‐GPU parallel platform, the robust optimization process can be completed in several minutes, which greatly improves the competitiveness of the proposed optimizer. In the future, we are going to extend the fast robust optimizer to compensate for the deformation of anatomical structures, respiratory movement, and so on.

## Conflicts of Interest

The authors have no relevant conflicts of interest to disclose.
